# Production of Mannosylerythritol Lipids Using Oils from Oleaginous Microalgae: Two Sequential Microorganism Culture Approach

**DOI:** 10.3390/microorganisms10122390

**Published:** 2022-12-02

**Authors:** Miguel Figueiredo Nascimento, Tiago Coelho, Alberto Reis, Luísa Gouveia, Nuno Torres Faria, Frederico Castelo Ferreira

**Affiliations:** 1 Department of Bioengineering, IBB—Institute for Biotechnology and Bioengineering, Instituto Superior Técnico, Universidade de Lisboa, Av. Rovisco Pais, 1049-001 Lisbon, Portugal; 2 Associate Laboratory i4HB—Institute for Health and Bioeconomy, Instituto Superior Técnico, Universidade de Lisboa, 1049-001 Lisbon, Portugal; 3 Laboratório Nacional de Energia e Geologia, I.P., Unidade de Bioenergia e Biorrefinarias, Estrada do Paço do Lumiar 22, 1649-038 Lisbon, Portugal; 4 GreenCoLab—Green Ocean Technologies and Products Collaborative Laboratory, CCMAR, Algarve University, 8005-139 Faro, Portugal

**Keywords:** oleaginous microalgae, bioprocess, mannosylerythritol lipids (MELs), *Moesziomyces* spp.

## Abstract

Mannosylerythritol lipids (MELs) are biosurfactants with excellent biochemical properties and a wide range of potential applications. However, most of the studies focusing on MELs high titre production have been relying in the use of vegetable oils with impact on the sustainability and process economy. Herein, we report for the first time MELs production using oils produced from microalgae. The bio-oil was extracted from *Neochloris oleoabundans* and evaluated for their use as sole carbon source or in a co-substrate strategy, using as an additional carbon source D-glucose, on *Moesziomyces* spp. cultures to support cell growth and induce the production of MELs. Both *Moesziomyces antarcticus* and *M. aphidis* were able to grow and produce MELs using algae-derived bio-oils as a carbon source. Using a medium containing as carbon sources 40 g/L of D-glucose and 20 g/L of bio-oils, *Moesziomyces antarcticus* and *M. aphidis* produced 12.47 ± 0.28 and 5.72 ± 2.32 g/L of MELs, respectively. Interestingly, there are no significant differences in productivity when using oils from microalgae or vegetable oils as carbon sources. The MELs productivities achieved were 1.78 ± 0.04 and 1.99 ± 0.12 g/L/h, respectively, for *M. antarcticus* fed with algae-derived or vegetable oils. These results open new perspectives for the production of MELs in systems combining different microorganisms.

## 1. Introduction

Nowadays, our planet is facing several climatic changes as a consequence of intensive use of fossil fuels. Such fossil fuels are mainly used to produce energy and chemicals improving human well-being and quality of life, but the process of their use results in carbon dioxide and other greenhouse gasses emissions, causing imbalance in the global ecosystem, and consequently, leading to increase of average plant temperatures [1]. One of the types of products more explored and used worldwide to support many human activities is surfactants, with a market value expected to reach 58.5 USD billion by 2027 and with a compound annual growth rate of 5.3% [2].

Surfactants are amphiphilic molecules that typically comprise an apolar tail and a polar head group and therefore they are able to interact simultaneously with polar and apolar compounds [3]. As a consequence of this unique characteristic, surfactants can adsorb at different interfaces, decrease surface tension between two phases, and promote different self-supported structures. These features make surfactants useful molecules for a wide range of applications, such as the formulations of cleaning agents, cosmetics, and pharmaceuticals, as well as the processing of leather, paper textiles, chemicals and food [4]. Nevertheless, surfactants are produced from petrochemicals, which are non-renewable compounds and their use contributes to greenhouse gas emissions. Moreover, the displacement of synthetic surfactants also poses a major threat to the environment due to their toxicity and their ability to increase other pollutants’ solubility. Such effects have significant impact due to the high persistence of synthetic surfactants on the ecosystem considering their capacity of adsorption into soils and their low biodegradability rate [5]. In this regard, microbial biosurfactants came out as an alternative to synthetic surfactants, given their higher biodegradability and lower toxicity [6]. Furthermore, microbial biosurfactants present different properties to their chemical counterpart and thus their potential to be used in different applications, such as therapeutic medicine and food preservatives, has been shown, as was well reviewed by Naughton et al. [7]. Currently, the market of microbial surfactants is led by glycolipids, such as sophorolipids (SLs), rhamnolipids (RMs) and mannosylerythritol lipids (MELs), and it is expected to reach 40 USD million by 2028 [8].

MELs, the biological products addressed in this study, are a family of glycolipids comprising a 4-O-β-D-mannopyranosyl-meso-erythritol moiety as the hydrophilic group and, typically, two fatty acids chains, as the hydrophobic group. The fatty acid chains are characterized by being relatively short, with a length of 8 to 12 carbons. MELs can be categorized into four main groups, depending on the number and position of the acetyl group within the D-mannose group. Namely, MEL-A is the di-acylated congener; MEL-B and MEL-D are the mono-acylated congeners, respectively, with the acetyl group in C6 and C4 of the D-mannose; and MEL-D is the congener with the D-mannose group deacylated [9]. MELs are one of the most promising microbial surfactants families, due to the low critical micellar concentration (CMC) with values as low as 0.0027 mM, a value 10-fold lower than the ones for SLs and RMs, where CMCs values are within the range of 0.12 to 0.30 mM [10,11,12,13]. Therefore, MELs potential uses have been reported in a wide range of applications in different fields, including hair and skin repair [14], the formulation of biopesticides [15] and of food preservatives [16] among others.

To this date, MELs have been mainly produced using hydrophobic substrates, such as soybean oil (SBO) or rapeseed oil (RO). The use of these substrates as carbon sources has been the dominant strategy to produce MELs as they support high growth of the producing yeasts and achieve productions of the glycolipid at high titres at values higher than 50 g/L. In fact, Rau et al. [9] achieved the highest productivity reported for MELs, at a value of 12 g/L/day in crude MELs, using as substrates a large amount of SBO, at a value around 186 g/L, D-glucose, at values of around 50 g/L and a mineral medium comprising 14 g/L of yeast extract and 16 g/L of sodium nitrate. However, the use of vegetable oils (SBO/RO) as main substrates increases the issues of process scale-up and represent a threat for food availability and prices. Moreover, the production of the vegetable crops from which such vegetable oil is sourced requires a large area of arable land for their production [17]. Therefore, more sustainable processes involving renewable residues, or substrates obtained from crops whose cultivation does not compete for agriculture land, are required for a sustainable and feasible MELs production on a meaningful scale.

**Figure 1:**
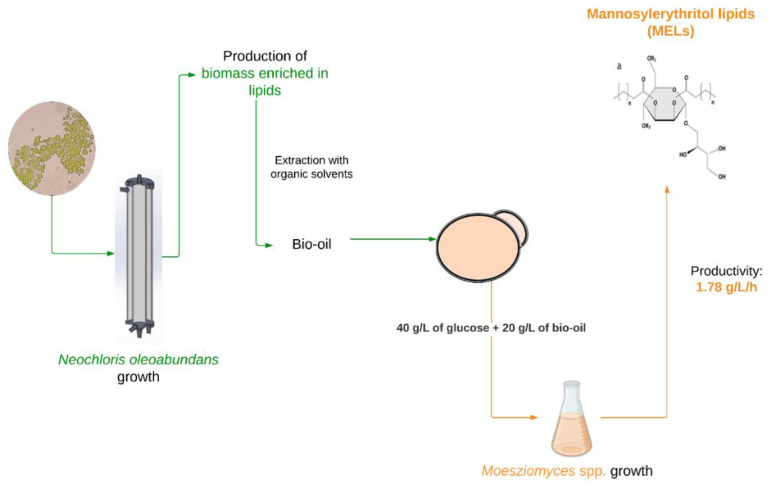
Schematic overview of MELs production from algae-derived bio-oils, produced by *Neochloris oleoabundans*.

To answer to this call, some studies attempted the replacement of SBO for waste frying oils (WFO), showing minimal impact on MELs productivity [18,19]. Nevertheless, the use of WFO as substrate for fermentations, depending on the source, previous intensity of use, and consequently level of oxidation and presence of inhibitory species can lead to different yeast cell growths and glycolipids productivity. Therefore, in MELs production using such oils as the main carbon source, it is important to address batch-to-batch variability of the WFOs [20]. Furthermore, for some applications, in particular to produce pharmaceuticals, cosmetics and food formulations, the use of more pure substrates is required. Therefore, in this study, with the aim to search for alternative sustainable hydrophobic substrates, we suggest the use of lipids derived from microalgae. In particular, the use of the oleaginous microalgae *Neochloris oleoabundans* came out as a promising candidate to produce such substrates, due to its capacity to accumulate high contents of intracellular lipids. Indeed, *N. oleoabundans* has been reported to accumulate up to 56% of biomass in lipid content for cultivations carried out without CO_2_ supplementation [21]. This microalgae has also been reported to be cultivated using industrial effluents, such as brewery effluents [22], which is interesting from the perspective of using residues as nitrogen sources. Figure 1 describes the strategy assessed on the current study, where, for the first time, the production of MELs using oils from oleaginous microalgae is investigated.

## 2. Materials and Methods

### 2.1. Microalgae Cultivation

The microalgae *Neochloris oleoabundans* #1185, obtained from the UTEX culture collection of the University of Texas, Austin, TX, USA, was used in this work. The stock culture was maintained with indirect sunlight in an Erlenmeyer shake flask, placed on the laboratory bench, and filled with 1/5 of working volume, corresponding to 50 mL of Bristol medium. This medium comprises 0.25 g/L of NaNO_3_, 0.175 g/L of KH_2_PO_4_, 0.075 g/L of K_2_HPO_4_; 0.075 g/L of MgSO_4_·7H_2_O; 0.075 g/L of MgSO_4_, 0.060 g/L of Fe-EDTA, 0.075 g/L of CaCl_2_, 0.025 g/L of NaCl, and 1 mL/L of trace elements, i.e., 2.860 g/L of H_3_BO_3_, 2.030 g/L of MnSO_4_·4H_2_O, 0.220 g/L of ZnSO_4_, 0.090 g/L of CoSO_4_·7H_2_O, 0.060 g/L of Na_2_MO_4_·2H_2_O, and 0.050 g/L of CuSO_4_. The stock cultures were renewed every two weeks. The bioreactors were started by adding 5 mL of stock culture as inoculum to an Erlenmeyer shake flask with 1/5 of working volume, corresponding to 50 mL volume. In other words, a 10% (V_inoculum_/V_culture medium_) of inoculum was used. The inoculated Erlenmeyer shake flask was incubated at 26 °C with an agitation of 130 rpm, with a light intensity of 80 µE/m^2^/s photosynthetic photon flux density (PPFD), in an illumination regime set to have 16 h of light and 8 h of dark over a day. The cultures were renewed with fresh culture medium every two weeks.

*Neochloris oleoabundans* was grown in two different bioreactors. Firstly, *N. oleoabundans* was grown in two home-made air bubble column bioreactors of 500 mL. Those experiments were carried out for characterization of the biomass, cell number, nitrate consumption and lipid formation over the cultivation period. Those microalgae cultivations were carried in duplicates, at 27 °C, with continuous agitation provided by bubbling filtered air set at a value of 1 vvm for 15 days under an illumination regime set to have 16:8 h of light/dark with a light intensity of 150 µE/m^2^/s PPFD on a light period. Then, for increase in biomass and to have more lipids to use in experiences, the microalgae were grown in larger 1 L glass bubble column bioreactors, where continuous agitation was achieved by bubbling filtered air at 0.9 VVM in Bristol medium at a temperature of 30 °C. While the nitrogen source was present on the medium, the CO_2_ was supplemented to obtain a biomass content on the range of 2 g/L. Once the microalgae reached this level of biomass, the cultivation was carried out for five additional days, but under nitrogen starvation and without supplementation of CO_2_. A final lipid content of 56% (DW) was achieved under such conditions. This first system was continuously illuminated by using six fluorescent lamps (Philips TL-DM 36W/54-765, Amsterdam, The Netherlands) with a light intensity of 150 µE/m^2^/s PPFD.

### 2.2. Yeast Strains, Substrate, and Cultivation Conditions

*Moesziomyces antarcticus* PYCC 5048^T^ and *Moesziomyces aphidis* PYCC 5535^T^ were obtained from the Portuguese Yeast Culture Collection (PYCC), Centro de Recursos Microbiológicos, Research Unit on Applied Molecular Biosciences at NOVA School of Science and Technology (CREM, UCBIO, FCT NOVA), Caparica, Portugal. Strains were plated in YM Agar (3 g/L of yeast extract, 3 g/L of malt extract, 5 g/L of peptone, 10 g/L of D-glucose and 20 g/L of agar) and incubated for 3 days at 30 °C. Stock cultures were prepared by the propagation of yeast cells in the liquid media, with similar composition to the one described below for use in inoculum preparation, after which they were stored in 20% (*v*/*v*) glycerol aliquots, at −70 °C. An inoculum was prepared by transferring the stocks cultures of *M. antarcticus* and *M. aphidis* into an Erlenmeyer flask with 1/5 working volume, corresponding to a volume of 50 mL of the medium. Such medium contains 0.3 g/L of MgSO_4_, 3 g/L of NaNO_3_, 0.3 g/L of KH_2_PO_4_, 1 g/L of yeast extract, 40 g/L of D-glucose. These cell cultures were incubated at 27 °C and kept at 250 rpm for 48 h. Then, 2.5 mL of this inoculum was added, corresponding to a ratio of 10% (*v*/*v*) of inoculum to culture volume, into an Erlenmeyer flask with 1/5 working volume, i.e., 25 mL of the cultivation medium. This medium was used for *Moesziomyces* media and contained 0.3 g/L of MgSO_4_, 3 g/L of NaNO_3_, 0.3 g/L of KH_2_PO_4_, 1 g/L of yeast extract, 40 g/L of D-glucose and 20 g/L of a hydrophobic source, which accordingly with the specific experimental condition was a different type of oil: (i) waste frying oils (WFO); or (ii) oils from *N. oleoabundans* (algae-derived bio-oils). As a control, a fermentation was also carried out, using 60 g/L of D-glucose as the only main carbon source, and with the culture media containing the salts and yeast extracted at the previously mentioned concentration. All cultures using oils were carried out in biological duplicates and incubated at 27 °C and kept at 250 rpm for 11 days.

### 2.3. Growth and Biomass Determination

Yeast and microalgae growth was estimated by measuring the cell dry weight (CDW), periodically, over the fermentation period. CDW was determined collecting 1 mL of culture broth, when it was centrifuged at 10,000× rpm for 6 min. The supernatant was discharged and the cell pellet washed with deionized water (twice) and dried at 60 °C for 48 h. Additionally, for microalgae cultivation, the concentration of cells per mL of culture was also quantified by counting the cells every day using a hemacytometer (LW scientific, Lawrenceville, GA, USA) and a microscope (Axiostar plus, Zeiss, Oberkochen, Germany). Briefly, a sample of cell cultivation broth was collected and diluted with an appropriate dilution factor (*Df*), then 15 µL of the resulting solution was added to the hemacytometer and cells on the 4 chambers were counted in duplicate and averaged. The cell density was estimated considering that each chamber has a volume of 10^−4^ mL,

(1) $$\frac{Cellnumber}{\mathrm{mL}}=\frac{\left(\frac{Cellcount}{4}\right)}{10^{-4}\mathrm{mL}}\times Df$$

### 2.4. Extraction of Oils from Microalgae

The extraction of bio-oils was carried out following the Bligh Dyer method, adapted by Araujo et al. [23]. *Neochloris oleoabundans* biomass was spray-dried (Christ Alpha 1-2 L0 plus) and an extraction was performed using methanol, chloroform, and water at a volume ratio of 5:3:1. The mixture was subjected to ultrasounds (Emmi-H30) for 40 min. After that, the biomass was separated by filtration. A solution of KCl at a concentration of 0.88% *w*/*v* was added to the liquid fraction in a peer-shaped separating funnel, and allowed to settle for 24 h. The bottom phase is recovered and evaporated in a rotary evaporator (Bucher) at 40 °C using 400 mbar, obtaining bio-oils. After evaporating, bio-oil was dissolved in hexane/ethyl acetate at a ratio 1:1 *v*/*v* and, again, the solvent was evaporated to remove traces of chloroform, and the bio-oil was recovered for further use.

### 2.5. MELs and Residual Lipids Quantification

During the fermentations, 1 mL of the culture broth samples was periodically taken and freeze-dried. The fatty acid content of the biological samples was determined by Gas Chromatography (GC) with a Flame Ionization Detector (FID), then transformed to methyl esters by methanolysis as described by Welz et al. [24]. In brief, 20 mL of pure methanol was cooled down to 0 °C under nitrogen atmosphere and 1 mL of acetyl chloride was added under stirring over 10 min to generate a water-free HCl/methanol solution. An internal standard solution comprised 4% (*v*/*v*) heptanoic acid and 96% (*v*/*v*) of n-hexane was prepared. The culture broth samples, after freeze-drying, were weighted and mixed with 2 mL of HCl/methanol solution and 100 µL of internal standard solution. These mixtures were incubated for 1 h at 80 °C for formation of the methyl esters. The resulting product was extracted with 1 mL of n-hexane and 1 mL of water. The organic phase was retrieved and 1 µL was injected in a GC system (Hewlett-Packard, HP5890, Palo Alto, CA, USA), equipped with a FID detector and an Agilent HP-Ultra2 capillary column (L 50 m × I.D. 0.32 mm, d_f_ 0.52 µm). The GC oven temperature was programmed to start from an initial plateau of 140 °C and increased up to 170 °C at a rate of 15 °C/min, then the temperature was further increased to 210 °C at a rate of 40 °C/min and then to 310 °C at a rate of 50 °C/min; there was a final isothermal plateau at 310 °C for 3 min. The carrier gas used was nitrogen, which was fed to the column using a split ratio of 1/25. MEL was quantified through the amount of methyl esters comprising fatty acid chains 8, 10 and 12 carbons long, as previously described [25].

### 2.6. Substrate Quantification

The quantification of D-glucose and nitrate was performed using high-performance liquid chromatography (HPLC). Culture broth samples were centrifuged at 10,000× rpm for 6 min, the supernatants were filtered through a 0.22 µm-pore size-filter and injected into the HPLC system (Merck Hitachi, Darmstadt, Germany) equipped with a refractive index detector (L-7490, Merck Hitachi, Darmstadt, Germany) for D-glucose quantification and a UV-VIS detector (L-2420 VWR Hitachi, Darmstadt, Germany) for sodium nitrate quantification. A Rezex ROA Organic Acid H+ column (300 mm × 7.8 mm, Phenomenex, Torrance, CA, USA) was fitted on the HPLC and operated at 65 °C. Sulfuric acid (5 mM) was used as the mobile phase at 0.5 mL/min. The quantification of D-glucose and sodium nitrate was performed using HPLC.

### 2.7. Statistical Analysis

Statistics were performed, using Graph-pad Prism 7 software by analysis of variance (two-way ANOVA) and estimations of *p*-values to evaluate the statistical significance of the differences between groups were corrected for simultaneous hypothesis testing according to Tukey’s method. The level of significance was set at *p* < 0.06.

## 3. Results and Discussion

### 3.1. Neochloris Oleoabundans Growth and Lipids Production

**Figure 2:**
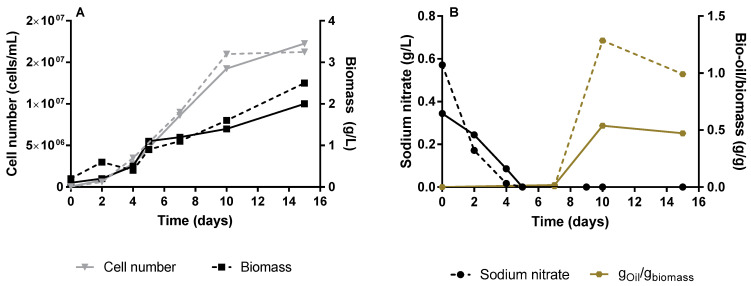
Cultivation of *Neocloris oleoabundans* #1185 in air-lift bioreactors for 15 days: cell number (inverted triangles) and biomass growth (squares) (**A**); sodium nitrate consumption (circles) and production of bio-oil per gram of biomass (**B**). Dashed and filled lines correspond to biological duplicates.

**Figure 3:**
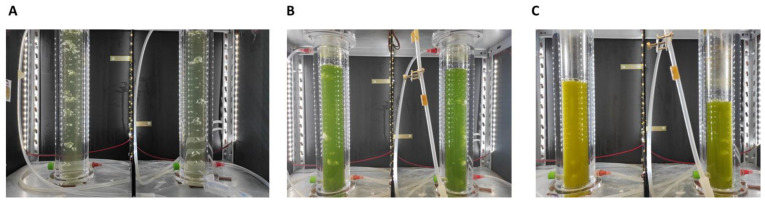
Images of *Neocloris oleoabundans* #1185 cultivation in air-lifts bioreactors at day 0 (**A**), 5 (**B**) and 15 (**C**) of fermentation.

*Neochloris oleoabundans* was initially grown in an air-lift bioreactor, over 15 days, without supplementation of CO_2_. Cell growth was monitored measuring the cell dry-weight (biomass) and counting the cell number (Figure 2A) during cultivation time. The nitrate consumption and production of bio-oil along the culture was also quantified (Figure 2B). The cell number and biomass grew continuously over the 15 days of culturing, with a cell number growth de-acceleration, while biomass continuously grew until 2.25 ± 0.25 g/L, after 10 days of cultivation. These data suggest that after day 10, the cells became larger and/or heavier. The production of lipids and/or carotenoids in microalgae strains is triggered by stress conditions, such as nutrient limitation or exposure to some physical factor (e.g., oxidative damage caused by light intensity, salt stress among others) [26]. In this study, the accumulation of intracellular lipids in *N. oleoabundans* was stimulated by the limitation of nitrogen source (sodium nitrate). Usually, as soon as the nitrogen source is consumed, the production of lipids by microalgae starts. However, in our experience, it is possible to observe a hiatus between the virtually complete depletion of sodium nitrate at day 5 of cultivation and the kick-off of intracellular lipids production, which starts at day 10. This can be explained by a metabolic delay associated with the conversion of NO_3_^−^, after entering the cell, into NO_2_^−^ by nitrate reductase, and follow up conversion of NO_2_^−^, after entering the chloroplast into NH_4_^+^ by the nitrite reductase, as reviewed by Salbitani et al. [27]. The production of lipids is noticed by a change in the color of the culture broth (Figure 3), which, after 10 days, changed from a green (Figure 3A,B) to a yellow/orange color (Figure 3C).

After 15 days of cultivation, a lipid cell content of 0.731 ± 0.259 g_bio-oil_/g_biomass_ was achieved, which is quite impressive, comparing to the result obtained by Li et al. [28]. However, in this study the authors achieved biomass and lipid productivities at values of 0.61 g/L/day and of 0.43 g/L/day, respectively. The values of biomass and lipids productivities obtained in the current study were 0.15 ± 0.016 g/L/day and 0.10 ± 0.026 g/L/day, respectively. While Li et al. [28] have used 5% of enriched CO_2_ on the reported *N. oleoabundans* cultures, the photosynthesis process reported on this study relies only on atmospheric CO_2_, which most probably delays and limits biomass and lipids production. Li et al. [28] also investigated the effect of the different nitrogen sources on the microalgae cultivation parameters, and concludes that the one that leads to higher lipid productivity was sodium nitrate when used at an optimal concentration of 0.84 g/L of sodium nitrate. Indeed, in the current study the sodium nitrate concentration was 3.4-fold lower than such optimal value, which can lead to lower biomass production and therefore to lower lipids productivity. These observations imply that the microalgae cultivation in the current study were still performed under sub-optimal conditions, and more studies are required to maximize lipids production by *N. oleoabundans*.

### 3.2. Production of MELs Using Algae-Derived Bio-Oils

After studying a production of lipids by *N. oleoabundans*, the capability of *Moesziomyce spp.* to produce MELs using the lipids (algae-derived bio-oils) produced by these microalgae was assessed. To obtain a higher amount of biomass and lipids, additional *N. oleoabundans* cultivations were performed on larger bioreactors of 1 L in the conditions described in Section 2.1. The produced intracellular lipids were extracted from spray-dried biomass, as described in Section 2.4, and the algae-derived bio-oils were obtained, as represented in Figure 1. The algae-derived bio-oils characterization (size of fatty acid chain) can be found in Table 1, with a profile similar to the one reported by Gouveia et al. [21]. Interestingly, when comparing algae-derived bio-oils and WFO fatty chain profile, one can observe that the former have more C16 chains, while WFO only have C18:0 fatty acid chain, which is in agreement with the reported values for acidic values, which are 3.3-fold higher for algae-derived bio-oils, at a value of 15.3 mgKOH [29], than the ones reported for WFO, at a value of 4.67 mg KOH [30]. Additional information concerning the type of fatty acids and their saturation/unsaturation properties can be found on Table 1.

**Figure 4:**
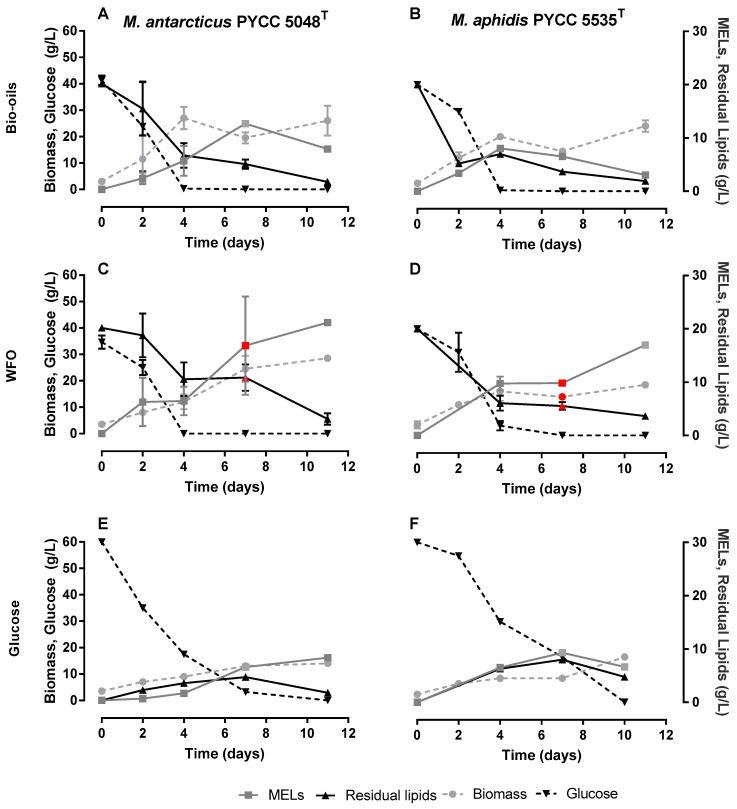
Cultivation of *M. antarcticus* PYCC 5048T (**A**,**C**,**E**) and *M. aphidis* PYCC 5535T (**B**,**D**,**F**) using D-glucose (40 g/L) and a hydrophobic source (20 g/L): Algae-derived bio-oil (**A**,**B**) and waste frying oil (**C**,**D**) or 60 g/L of D-glucose (**E**,**F**) with no further addition of hydrophobic carbon source. The points in red indicate the presence of beads enriched in MELs in the media. Standard deviations values lower than 1 g/L are not represented.

As previously described, MELs are mainly produced using hydrophobic substrates, such as SBO, as the main carbon sources. This is an efficient strategy to reach high MELs titres, but usually also results in low purities, resulting from unconsumed substrates. On the other hand, the use of hydrophilic carbon sources, such as D-glucose, allows achieving high MELs purities, but the MELs titres obtained are low, as observed by Faria et al. [25] on *Moesziomyces* spp. cultivations using D-glucose, D-xylose and L-arabinose. Previous studies by the authors show the advantages of following a co-substrate strategy that employs both hydrophilic (D-glucose) and hydrophobic (oils) carbon sources, namely the ability to simultaneously boost MELs titres and purities [31]. Therefore, in the current study, a similar co-substrate cultivation strategy was followed. Following such a co-substrate strategy, three different conditions were performed for *M. antarcticus* (Figure 4A,C,E) and *M. aphidis* (Figure 4B,D,F). Two of these cultivations have started with 40 g/L of glucose and 20 g/L of a hydrophobic carbon source, where the hydrophobic carbon source was algae-derived bio-oils (Figure 4A,B) or WFO (Figure 4C,D). The third strategy included the use of D-glucose as the sole carbon source (Figure 4E,F), but using 60 g/L of D-glucose in order that this condition was fed with a similar carbon molar equivalent to the assays that follow a co-substrate strategy. The results are summarised in Table 2 and Figure 4.

When analysing the patterns of substrate consumption, it is possible to observe that the consumption rate of D-glucose is slightly higher when algae-derived bio-oil is used instead of WFO (19 and 7% for *M. antarcticus* and *M. aphidis*, respectively). Importantly, the consumption of D-glucose in the presence of either of the oils is relatively high, being virtually depleted at day 4 of the fermentation, which indicates that there is no catabolic repression of oils on D-glucose consumption. For the experiment using D-glucose alone, a higher concentration of this substrate was used and a slightly lower rate of D-glucose was observed. The data also suggest that the consumption of oil is faster for algae-derived bio-oil than for WFO. This pattern can be explained by the composition of the bio-oil, which has an acidic value of 15.3 mg KOH [29], 3.3-fold higher than the value for WFO (4.67 mg KOH) [30]. This means that the algae-derived bio-oil has a higher content in free fatty acids and monoacylglycerides or diacylglycerides, while WFO is richer in triacylglycerides. In presence of a hydrophobic substrate, *Moesziomyces* spp. have the ability to produce lipases for breakdown of triacylglycerides into free fatty acids to be assimilated by the cell [32]. However, feeding the fermentation with a substrate already partially broken down can speed up its assimilation and the incorporation of the lipidic molecules into MELs, which will be metabolized through the chain-shortening pathway or partial β-oxidation [33]. Therefore, the use of lipids from *N. oleoabundans* with higher acidic value and more free fatty acids can lead to a faster substrate consumption and higher MELs productivities.

The faster consumption of algae-derived bio-oil led to a maximum MELs titre of 12.47 ± 0.28 (Day 4) and 5.72 ± 2.32 g/L (Day 7) for *M. antarcticus* and *M. aphidis*, respectively. The maximum MELs titre was obtained earlier for *Moesziomyces* spp. cultivations using algae-derived bio-oils than for the ones using WFO as carbon source (day 11 for both strains). Remarkably, there was no significant difference in maximum productivity observed for cultures based on algae-derived bio-oils and WFO, respectively, with values of 1.78 ± 0.04 g/L/h (algae-derived bio-oils) and 1.99 ± 0.12 g/L/h (WFO) for *M. antarcticus* or 1.43 ± 0.58 g/L/h (algae-derived bio-oils) and 1.54 ± 0.01 g/L/h (WFO) for *M. aphidis*. However, the final titres were 2.86 and 5.57-fold higher for WFO-based fermentation than for the ones using bio-oils from microalgae. Namely, when WFO was used, the MELs maximum titres were 21.94 ± 1.31 g/L and 16.98 ± 0.39 g/L, respectively, for *M. antarcticus* and *M. aphidis* cultures. The high discrepancy on maximum MELs titres could be related with the potential consumption of MELs after day 7, due to the low contents in algae-derived bio-oil, which calls for further fermentation optimization using bio-oils feed batch strategies.

Overall, MELs titres and productivities under all tested conditions were higher when *M. antarcticus* was used rather than *M. aphidis*, and this may be due to the capacity of *M. aphidis* to create reserves of free fatty acids. In fact, this phenomenon is observed when D-glucose is used as the sole carbon source, where the low MELs titres and higher yeast lipids accumulation led to a final purity of 58%.

**Figure 5:**
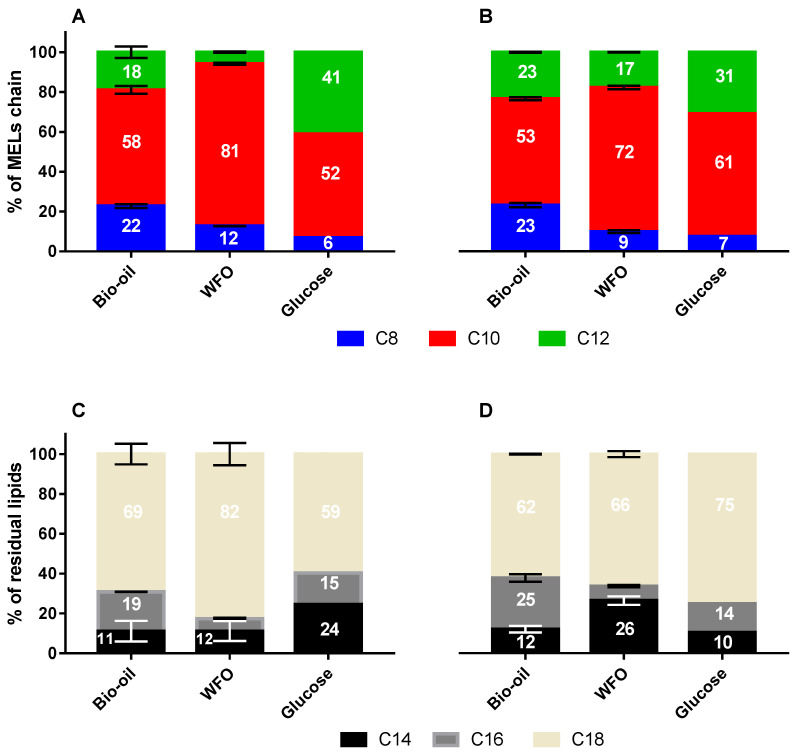
Type of fatty acids chains in maximum MELs titres (C8, C10, C12) and residual lipids (C14, C16, C18) produced by *M. antarcticus* PYCC 5048T (**A**,**C**) and *M. aphidis* PYCC 5535T (**B**,**D**) with different substrates used: (1) Bio-oils and D-glucose; (2) Waste frying oils and D-glucose and (3) D-glucose alone. Standard deviations values lower than 1% are not represented.

The WFO and the algae-derived bio-oils have very different compositions; while the former is richer in triacylglycerides and C18 carbon chains, the latter have higher contents in free fatty acids and C16 unsaturated chains. Therefore, it was investigated whether the type of oil used would affect the chains present on the MELs produced using the different carbon sources (Figure 5). Interestingly, the results show that when microalga’s bio-oil is used, for both strains, the content of C8 chains in MELs is 2-fold higher when MELs is produced from WFO or D-glucose. This result is consistent with the algae-derived bio-oils composition, richer on shorter carbon chains, and the hypothesis of partial β-oxidation of free fatty acids fed to the fermentation, followed by their integration on MELs molecules upon their biosynthesis [33]. The profiles of lipidic MELs chains in the cultures using D-glucose alone are consistent with the hypothesis that lipids to be integrated into the MELs follow the canonical “de-novo” synthesis of fatty acids chains up to C18, which are to be incorporated before into mannose and erythritol and need to follow the partial β-oxidation route.

The production of MELs using lipids produced by other microorganisms has been previously reported in the literature, but using oleaginous yeasts, instead of microalgae, for bio-oils production. Namely, Akkermans et al. [34] have used lipids produced from *Cutaneotrichosporon oleaginous* as a carbon source for *M. aphidis* and a comparative overview of results from that study and the results obtained here in the presented work can be found on Table 3. The final MELs titre obtained was 2.4-fold lower than the one obtained in the current study. However, the authors have used *Cutaneotrichosporon oleaginous* cells lysate, obtained by mechanic pre-treatment, as the carbon source in *Moesziomyces* spp. cultivations. Such a strategy includes additional energy needed to obtain the cell lysate but does not rely on the intensive use of organic solvents for lipids extraction. Therefore, further studies using lysates of *Neochloris oleoabundans* mechanically obtained might be also relevant.

## 4. Conclusions

This study reports, for the first time, on the production of MELs from microalgae-derived oil. When using the same strain, no significant differences were noticed on MELs productivities for conditions using bio-oil or waste frying oils. This study points out an alternative route for the research and design of bioprocesses using a more sustainable class of bio-oils for MELs production. Still, there is a call for new and more sustainable approaches to extract lipids from microalgae avoiding the use of organic solvents, such as a high-pressure homogenizer. Furthermore, the current study suggests that a fed-batch fermentation can be used to optimize MELs production and overcome MELs titres limitations driven from the fast consumption of algae’s bio-oils. Finally, the current study illustrates that the properties of different substrates can influence the produced MELs congeners. The algae-derived bio-oils, which comprise a lipid mixture with higher C16 and lower C18 fatty acids content than WFO, promoted the production of MELs mixture with high higher content of smaller C8 lipidic chains.

## Figures and Tables

**Table 1:** Size of fatty acid chain (%) for algae-derived bio-oils and waste frying oils (WFO).

Fatty Acid Chain (%)	Algae-Derived Bio-Oils	WFO
C14:0	-	-
C16:0	12.66	0.13
C16:1	8.88	0
C18:0	59.84	95.43
C18:1	18.62	4.45

**Table 2:** Rate of D-glucose consumption (Rs); maximum biomass produced; Maximum MEL yield obtained (g/L); yield of MELs produced (gMELs/gSubstrate), maximum productivity (g/L/h) and purity (g/g) for *M. antarcticus* PYCC 5048T and *M. aphidis* PYCC 5535T using 40 g/L of D-glucose with 20 g/L of hydrophobic carbon source (algae-derived bio-oils or waste frying oils).

Parameters	*M. antarcticus* PYCC 5048^T^			*M. aphidis* PYCC 5535^T^		
	Algae-Derived Bio-Oils	WFO	D-Glucose	Algae-Derived Bio-Oils	WFO	D-Glucose
Rs (g/L/h)	0.43 ± 0.02	0.36 ± 0.02	0.31	0.41 ± 0	0.38 ± 0.01	0.36
Biomass_max_ (g/L)	27.0 ± 3.0 (Day 4)	28.5 ± 0.5 (Day 11)	17	24.5 ± 1.5 (Day 11)	28.5 ± 0.5 (Day 11)	14
MEL_max_ (g/L)	12.47 ± 0.28 (Day 7)	21.94 ± 1.31 (Day 11)	8.09 (Day 11)	5.72 ± 2.32 (Day 4)	16.98 ± 0.39 (Day 11)	6.64 (Day 11)
Y _MEL/Substrate_ (g/g)	0.21 ± 0.01	0.37 ± 0.02	0.13	0.1 ± 0.04	0.28 ± 0.39	0.11
Productivity_max_ (g/L/day)	1.78 ± 0.04	1.99 ± 0.12	0.73	1.43 ± 0.58	1.54 ± 0.01	0.60
MEL purity (g/g)	0.84 ± 0.01	0.88 ± 0.03	0.84	0.61 ± 0.06	0.88 ± 0.04	0.58

Biomass_max_–maximum biomass cell dry weight (g/L); rs–sugar consumption rate (g/L/h); MELs_max_–maximum MEL produced (g/L); Y MELs/Substrate consumed–maximum MEL yield (g/g); Productivity_max_–Maximum productivity (g/L/h); MELs purity (g/g) at the end of the fermentation-Ratio of g of MELs to the sum of g of MELs and residual lipids.

**Table 3:** Summary of the results obtained by Akkermans et al. [34] and the results here presented, that report on the use of two sequential microorganisms for the production of MELs, including type of strain, type of product, titre, yield and productivity. * Values are not present in the article and are calculated by us.

Bio-Oil Producing Strain	Product Recovery	MELs Producing Strain	MELs Max (g/L)	Yield (gMELs/gsubstrate)	Productivity (g/L/h)	Ref
*Cutaneotrichosporon oleoginosus*	Use of cell lysates	*M. aphidis*	2.3	0.19 *	0.5 *	Akkermans et al. [34]
*Neochloris* *oleoabundans*	Use or organic solvents	*M. aphidis*	5.72 ± 2.32	0.1 ± 0.04	1.43 ± 0.58	This study
		*M. antarcticus*	12.47 ± 0.28	0.21 ± 0.01	1.78 ± 0.04	

## Data Availability

The data supporting the conclusions of this article are included within the article and its figures and there was no large datasets generated or analysed during the current study.

## References

[1] Dai A. (2011). Drought under Global Warming: A Review. Wiley Interdiscip. Rev. Clim. Chang..

[2] Dixit S., Danekar R., Prasad E. Surfactants Market Size, Share | Industry Analysis & Forecast, 2027. https://www.alliedmarketresearch.com/surfactant-market.

[3] Kronberg B., Holmberg K., Lindman B. (2014). Types of Surfactants, Their Synthesis, and Applications. Surf. Chem. Surfactants Polym..

[4] Rodrigues L., Banat I.M., Teixeira J., Oliveira R. (2006). Biosurfactants: Potential Applications in Medicine. J. Antimicrob. Chemother..

[5] Ying G.-G. (2006). Fate, Behavior and Effects of Surfactants and Their Degradationproducts in the Environment. Environ. Int..

[6] Marchant R., Banat I.M. (2012). Biosurfactants: A Sustainable Replacement for Chemical Surfactants?. Biotechnol. Lett..

[7] Naughton P.J., Marchant R., Naughton V., Banat I.M. (2019). Microbial Biosurfactants: Current Trends and Applications in Agricultural and Biomedical Industries. J. Appl. Microbiol..

[8] MarketWatch. Microbial Biosurfactants Market Share. https://www.marketwatch.com/press-release/microbial-biosurfactants-market-share-size-2022-global-companies-consumption-drivers-top-leading-countries-trends-growth-factors-forces-analysis-revenue-challenges-and-global-forecast-2028-2022-02-16.

[9] Rau U., Nguyen L.A., Roeper H., Koch H., Lang S. (2005). Fed-Batch Bioreactor Production of Mannosylerythritol Lipids Secreted by Pseudozyma Aphidis. Appl. Microbiol. Biotechnol..

[10] Lang S., Wullbrandt D. (1999). Rhamnose Lipids—Biosynthesis, Microbial Production and Application Potential. Appl. Microbiol. Biotechnol..

[11] Konoshi M., Fukuoka T., Morita T., Imura T., Kitamoto D. (2008). Production of New Types of Sophorolipids by Candida Batistae. J. Oleo Sci..

[12] Imura T., Ohta N., Inoue K., Yagi N., Negishi H., Yanagishita H., Kitamoto D. (2006). Naturally Engineered Glycolipid Biosurfactants Leading to Distinctive Self-Assembled Structures. Chemistry.

[13] Kitamoto D., Isoda H., Nakahara T. (2002). Functions and Potential Applications of Glycolipid Biosurfactants—From Energy-Saving Materials to Gene Delivery Carriers. J. Biosci. Bioeng..

[14] Morita T., Fukuoka T., Imura T., Kitamoto D. (2013). Production of Mannosylerythritol Lipids and Their Application in Cosmetics. Appl. Microbiol. Biotechnol..

[15] Fukuoka T., Yoshida S., Nakamura J., Koitabashi M., Sakai H., Abe M., Kitamoto D., Kitamoto H. (2015). Application of Yeast Glycolipid Biosurfactant, Mannosylerythritol Lipid, as Agrospreaders. J. Oleo Sci..

[16] Shu Q., Wei T., Lu H., Niu Y., Chen Q. (2020). Mannosylerythritol Lipids: Dual Inhibitory Modes against Staphylococcus Aureus through Membrane-Mediated Apoptosis and Biofilm Disruption. Appl. Microbiol. Biotechnol..

[17] Anto S., Mukherjee S.S., Muthappa R., Mathimani T., Deviram G., Kumar S.S., Verma T.N., Pugazhendhi A. (2020). Algae as Green Energy Reserve: Technological Outlook on Biofuel Production. Chemosphere.

[18] Niu Y., Wu J., Wang W., Chen Q. (2019). Production and Characterization of a New Glycolipid, Mannosylerythritol Lipid, from Waste Cooking Oil Biotransformation by *Pseudozyma aphidis* ZJUDM34. Food Sci. Nutr..

[19] Nascimento M.F., Barreiros R., Cristina A., Frederico O., Ferreira C., Faria N.T. (2022). Moesziomyces spp. Cultivation Using Cheese Whey: New Yeast Extract-Free Media, β-Galactosidase Biosynthesis and Mannosylerythritol Lipids Production. Biomass Convers. Biorefin..

[20] Bhuiya M.M.K., Rasul M.G., Khan M.M.K., Ashwath N., Azad A.K. (2016). Prospects of 2nd Generation Biodiesel as a Sustainable Fuel—Part: 1 Selection of Feedstocks, Oil Extraction Techniques and Conversion Technologies. Renew. Sustain. Energy Rev..

[21] Gouveia L., Evangelista A., Lopes T., Reis A. (2009). Neochloris Oleabundans UTEX # 1185: A Suitable Renewable Lipid Source for Biofuel Production. J. Ind. Microbiol. Biotechnol..

[22] Ferreira A., Ribeiro B., Ferreira A.F., Tavares M.L.A., Vladic J., Vidović S., Cvetkovic D., Melkonyan L., Avetisova G., Goginyan V. (2019). Scenedesmus Obliquus Microalga-Based Biorefinery—From Brewery Effluent to Bioactive Compounds, Biofuels and Biofertilizers—Aiming at a Circular Bioeconomy. Biofuels Bioprod. Biorefin..

[23] Araujo G.S., Matos L.J.B.L., Fernandes J.O., Cartaxo S.J.M., Gonçalves L.R.B., Fernandes F.A.N., Farias W.R.L. (2013). Extraction of Lipids from Microalgae by Ultrasound Application: Prospection of the Optimal Extraction Method. Ultrason. Sonochem..

[24] Welz W., Sattler W., Leis H.J., Malle E. (1990). Rapid Analysis of Non-Esterified Fatty Acids as Methyl Esters from Different Biological Specimens by Gas Chromatography after One-Step Esterification. J. Chromatogr. B Biomed. Sci. Appl..

[25] Faria N.T., Santos M.V., Fernandes P., Fonseca L.L., Fonseca C., Ferreira F.C. (2014). Production of Glycolipid Biosurfactants, Mannosylerythritol Lipids, from Pentoses and d-Glucose/d-Xylose Mixtures by Pseudozyma Yeast Strains. Process Biochem..

[26] Sun X.M., Ren L.J., Zhao Q.Y., Ji X.J., Huang H. (2018). Microalgae for the Production of Lipid and Carotenoids: A Review with Focus on Stress Regulation and Adaptation. Biotechnol. Biofuels.

[27] Salbitani G., Carfagna S. (2021). Ammonium Utilization in Microalgae: A Sustainable Method for Wastewater Treatment. Sustainability.

[28] Li Y., Horsman M., Wang B., Wu N., Lan C.Q. (2008). Effects of Nitrogen Sources on Cell Growth and Lipid Accumulation of Green Alga Neochloris Oleoabundans. Appl. Microbiol. Biotechnol..

[29] Gouveia L., Janelas J., Tropecêlo A.I., Oliveira A.C. (2016). Microalga Nannochloropsis sp. Biomass for Biodiesel Production: Conventional (Cell Disruption) and in Situ Transesterification. J. Mar. Biol. Oceanogr..

[30] Robles Arévalo A. (2015). Substrates for the Sustainable Production of Mannosylerythritol Lipids: Biological Oils vs. Nanofiltrated Lignocellulosic Hydrolysates. Master’s Thesis.

[31] Faria N.T., Nascimento M.F., Ferreira F.A., Esteves T., Santos M.V., Ferreira F.C. (2022). Efficient Production of Mannosylerythritol Lipids by Moesziomyces Spp.: Substrates of Opposite Polarities for Optimal Biosurfactant Production and Downstream Processing. Appl. Biochem. Biotechnol..

[32] Ueda H., Mitsuhara I., Tabata J., Kugimiya S., Watanabe T., Suzuki K., Yoshida S., Kitamoto H. (2015). Extracellular Esterases of Phylloplane Yeast Pseudozyma Antarctica Induce Defect on Cuticle Layer Structure and Water-Holding Ability of Plant Leaves. Appl. Microbiol. Biotechnol..

[33] Kitamoto D., Yanagishita H., Haraya K., Kitamoto H.K. (1998). Contribution of a Chain-Shortening Pathway to the Biosynthesis of the Fatty Acids of Mannosylerythritol Lipid (Biosurfactant) in the Yeast Candida Antarctica: Effect of β-Oxidation Inhibitors on Biosurfactant Synthesis. Biotechnol. Lett..

[34] Akkermans V., Verstraete R., Braem C., D’Aes J., Dries J. (2020). Mannosylerythritol Lipid Production from Oleaginous Yeast Cell Lysate by Moesziomyces Aphidis. Ind. Biotechnol..

